# A Cross-Sectional Study of Coronavirus Disease Prevention Practices among University Staff and Students in Durban, South Africa in 2020–2021

**DOI:** 10.3390/idr15010009

**Published:** 2023-01-23

**Authors:** Maureen Nokuthula Sibiya, Kelechi Elizabeth Oladimeji, Felix Emeka Anyiam, Olanrewaju Oladimeji

**Affiliations:** 1 Division of Research, Innovation and Engagement, Mangosuthu University of Technology, Umlazi 4031, South Africa; 2 Department of Public Health, Faculty of Health Sciences, Walter Sisulu University, Mthatha 5099, South Africa; 3 Department of Laboratory Medicine Pathology, Faculty of Health Sciences, Walter Sisulu University, Mthatha 5099, South Africa; 4 Faculty of Health Sciences, Durban University of Technology, Durban 4001, South Africa

**Keywords:** COVID-19 prevention, practices, University Staff and Students, Durban University of Technology, South Africa

## Abstract

**Background:** Globally, the COVID-19 pandemic has had a negative impact on individuals, education, and the economy. During its peak, the pandemic forced school closures. Although there is currently no cure for corona virus, non-pharmaceutical measures can help prevent its spread. Among these preventive measures are regular handwashing with soap and water or the use of hand sanitizers, avoiding touching the mouth, nose, and eyes, social distancing, and the use of face masks. As a result, this study investigated COVID-19 prevention practices among Durban University of Technology staff and students in South Africa. **Methods:** Using a cross-sectional study design, data were gathered online via self-administered, structured questionnaires from 5849 university students and staff members between May 2020 and March 2021. Utilizing descriptive statistics, the characteristics of the study sample were reported. Using logistic regression models, the relationship between demographic characteristics and the overall level of COVID-19 preventive practices was evaluated. **Results:** The multivariate logistic regression model showed statistically significantly associations for COVID-19 preventive practices by: male (AOR: 9.815, 95% CI: 1.721–55.959, *p* = 0.01) compared to female participants, single participants (AOR: 6.012, 95% CI: 2.070–17.461, *p* = 0.001) compared to other marital categories, and those in the faculty of Health Sciences (AOR: 1.721, 95% CI: 1.023–2.894, *p* = 0.041) compared to other faculties. **Conclusions:** Overall, the study’s preventive practices were commendable; they were also influenced by socio-demographic factors such as age, gender, marital status, and university faculty. Increasing age was associated with reduced compliance with COVID-19 preventive practices. In addition, men demonstrated greater caution than women.

## 1. Introduction

The COVID-19 pandemic is the fifth pandemic and comes 100 years after the last, in 1918 [1,2]. It started with unusual cases of pneumonia in Wuhan, Hubei Province, China in December 2019 [1]. The novel viral agent responsible for the possibly infectious disease of the century was later named Severe Acute Respiratory Syndrome Corona Virus-2 (SARS-CoV-2) and the disease known as Corona virus disease (COVID-19) [3,4]. Around the time it was declared a global pandemic in March 2021 [5], more than 120 million cases with over 2.6 million mortalities had been reported globally [6,7]. Africa accounted for about 20,313 (0.84%) of global cases, with South Africa having the highest number of confirmed cases 3158 (15.56%) in the African continent, followed by Egypt 3144 (15.48%) as of 20 April 2020 [7,8]. The first case of COVID-19 in South Africa was reported on the 5 March 2020 [9], since then the country had over 1.5 million positive tests and 51,634 deaths recorded, with a recovery rate of 95% as of 17 March 2021 [10]. Evidence indicates that human-to-human transmission is through droplets from coughing or sneezing or direct contact with infected surfaces which may result in cough or/and fever or/and sore throat as the main symptoms within a week of contracting the viral infection [2]. 

As a response to the deadly global COVID-19 pandemic, countries around the world took a variety of measures to stop the spread of this virus and lessen its negative impact [11]. Non-pharmaceutical interventions (NPIs) include promoting a high level of hygiene (e.g., frequent handwashing with soap), temporary closure of educational institutions, museums, theaters, some shopping malls and restaurants, and suspension of religious activities and public gatherings [12]. Despite the availability of vaccines, the most effective preventative measures include handwashing with soap and water or using hand sanitizers, avoiding touching the mouth, nose, and eyes, social isolation, and wearing a face mask. [13]. As the global fight against COVID-19 continues and plans for alternative forms of education are developed, it is crucial that university students and faculty adhere to current preventive measures, such as handwashing and social distancing. This is due to the fact that knowledge, attitudes, and preventive practices (KAP) regarding COVID-19 also play a crucial role in this fight [6]. Therefore, the purpose of this study was to investigate the COVID-19 preventive practices of faculty and students at Durban University of Technology (DUT), South Africa. The findings will indicate the level of compliance with COVID-19 infection prevention practices in higher education institutions to inform evidence-based policy on additional strategies to be implemented by relevant health and education stakeholders.

## 2. Methods

**Study site/design:** This is a cross-sectional study that was conducted in DUT, South Africa. The university is in the top five of all universities in the country and has over 33,000 students in all its campuses situated in both Durban and Pietermaritzburg [14]. It also located in KwaZulu-Natal province, where there are over nine (8) public higher institutions including DUT [15].

**Study population and sampling**: Applying a convenient sampling method, all consenting staff and students in DUT were eligible to participate in the study. 

**Data collection and management:** An online semi-structured questionnaire was designed using Google forms and piloted. The questionnaire consisted of questions on demographics, position in DUT and preventive practices towards COVID-19 transmission. After necessary revision from the pilot exercise, data was collected from consenting staff and students at the university through self-administration. Data management involved exporting data from Google forms to MS Excel for cleaning and further analysis with Statistical Package for Social Sciences (SPSS) version 20.0. 

**Data analysis:** Analysis involved both descriptive statistics to report the characteristics of the study sample and inferential statistics (logistic regression). In measuring the overall level of preventive practices of COVID-19 transmission among participants, nine (8) preventive practice questions were asked, and scoring was done based on a poor and good/adequate answer methodology. The scores were summed for all participants. The overall good/adequate preventive practices of COVID-19 transmission were scored from 6–9 and poor preventive practices of COVID-19 transmission were scored ≤5. Bivariate and multivariate logistic regression models were used to assess the association between demographic characteristics and overall preventive practices of COVID-19 among the study participants.

## 3. Results

### 3.1. Demographics of Participants 

The study participants included 3351 females (57.29%), 2495 males (42.61%), and six “others” (0.10%) who did not provide information about their gender. The mean age was 23.67 ± 6.47. The highest percentage, 80.55% (4729), was found among those aged 17–26; the second highest percentage, 14.57% (852), was found among those aged 27–36. The lowest percentage, 4.59% (268), was of those within the age range of 37 and more (Table 1). The vast majority of respondents (94.28%; 5489) were single, while only 4.55% were married (265). The biggest percentage of participants (56.95%) were those with a university education (331), followed by those with a secondary/high school education (42.1%; 2469), those with an elementary education (0.51%; 30), and those with no formal education (0.32%; 19).

The majority of the respondents were students, 96.14% (562), located across the seven campuses of the university, and with more than 75% located in three of the campuses: Ritson, ML Sultan and Steve Biko Campus. They were from the faculties of Management Sciences, 30.19% (1766), Accounting and Informatics, 22.77% (1332), Engineering and the Built Environment, 18.19% (194), while Arts & Design, Applied Sciences and Health Sciences accounted for the rest. The university accommodation facility housed 29.66% (1735), while 70.34% (4116) lived outside the university, as shown in Table 2. 

### 3.2. Preventive Practices of COVID-19 Transmission 

Twenty-five percent of respondents (1501) reported visiting a busy area within the previous week; 97.97% (5730) wore facemasks; 40.50% (2369) wore gloves; 98.07% (5736) used hand sanitizer; and 94.61% (5534) routinely cleaned their hands after contacting surfaces. Those who kept their hands away from their mouths, eyes, and noses made up 87.86% (5139); those who covered their noses with tissue paper or cloth when they sneezed in public or at home, 94.58% (5532); those who observed appropriate social distance when in public spaces, 97.11% (5680); and 73.73% of respondents said they would accept the COVID-19 vaccine if it were developed (4249) (as shown in Table 3). Overall, the highest proportion (93.79%) adhered to good/adequate preventive practices against COVID-19 (Table 4).

### 3.3. Bivariate Logistic Regression Model

The bivariate logistics regression model showed statistically significant associations with preventive practices of COVID-19 transmission for the following variables: Gender, Age, Marital Status and Faculty. No statistically significant associations were observed with Position at DUT and educational level status (Table 5). Study participants that are within the age groups 27–36 years (OR: 9.342, 95% CI: 1.555–56.111, *p* = 0.015), 37–46 years (OR: 13.897, 95% CI: 2.257–85.586, *p* = 0.005), 45–56 years (OR: 22.00, 95% CI: 2.983–162.266, *p* = 0.002), 57–66 years (OR: 41.333, 95% CI: 2.875–594.144, *p* = 0.006) and ≥ 67 years (OR: 19.333, 95% CI: 1.327–281.597, *p* = 0.030) showed a statistically significant increased likelihood for having good/adequate preventive practices of COVID-19 transmission, with those within the age groups 47–56 and 57–66 years having the strongest relationship with preventive practices of COVID-19 transmission (Table 5). 

Male (OR: 0.099, 95% CI: 0.018–0.544, *p* = 0.008), and female study participants (OR: 0.558, 95% CI: 0.450–0.692, *p* = 0.001), showed a statistically significant reduced likelihood of having good/adequate preventive practices of COVID-19 transmission, with male participants having a more reduced likelihood than female participants (Table 5). Moreover, single (OR: 0.204, 95% CI: 0.076–0.551, *p* = 0.002) and married study participants (OR: 0.464, 95% CI: 0.228–0.946, *p* = 0.035), showed a statistically significant reduced likelihood for having good/adequate preventive practices of COVID-19 transmission, with single participants having a more reduced likelihood than married participants (Table 5).

Participants in the Faculties of Engineering & the Built Environment (OR: 0.606, 95% CI: 0.383–0.959, *p* = 0.032), Arts & Design (OR: 0.543, 95% CI: 0.341–0.865, *p* = 0.01), Applied (OR: 0.579, 95% CI: 0.348–0.964, *p* = 0.035) and Health Sciences (OR: 0.526, 95% CI: 0.315–0.879, *p* = 0.014) showed a statistically significant reduced likelihood for having good/adequate preventive practices of COVID-19 transmission, with participants in the Health Sciences having the most reduced likelihood (Table 5).

### 3.4. Multivariate Logistic Regression Model

After adjustment for other factors in the model, the multivariate logistic regression model showed statistically significantly higher odds for gender, with male participants (AOR: 9.815, 95% CI: 1.721–55.959, *p* = 0.01) having an eight-times stronger relationship with preventive practices of COVID-19 transmission than female participants (AOR: 1.692, 95% CI: 1.356–2.112, *p* = 0.001), single participants (AOR: 6.012, 95% CI: 2.070–17.461, *p* = 0.001), and those in the Faculty of Health Sciences (AOR: 1.721, 95% CI: 1.023–2.894, *p* = 0.041) as shown in Table 6.

The variable age, after adjusting for confounding variables, showed statistically significantly lower odds with preventive practices of COVID-19 transmission in all age groups, as shown in Table 6. The Faculties of Engineering & the Built Environment, Arts & Design and Applied Sciences were no longer statistically significant in the multivariate logistic regression, and thus were confounding determinants (Table 6).

## 4. Discussion

The majority of respondents were single females. Very few of the total study population were university staff or both staff and students. In other comparable studies conducted in Egypt, China, and Jordan, the higher proportion of females ranged from 61 to 70% [16,17,18,19], compared to 57% in the present study. In addition, approximately four out of five respondents were between the ages of 17 and 26, with an average age of 23.67 years. This is comparable to the characteristics of the study population described by Peng et al. [18], in which the average age of Chinese undergraduates ranged from 17 to 25 years, with a mean age of 23 years. In addition, only fifty percent of respondents held a bachelor’s degree or higher as their highest level of education. The study revealed that the vast majority of respondents observe positive, correct, and adequate COVID-19 prevention practices. This is consistent with numerous studies conducted in universities across the globe, among healthcare professionals and the general public [19,20,21,22,23,24]. Contradictory results were reported in a study conducted among students at two Pakistani universities where inadequate preventive measures were observed [25]. This study’s commendable finding regarding good preventive practices is indicative of the university community’s eagerness to make necessary behavioral changes for their own and the public’s health. In addition, it may suggest that it is the result of the South African government’s information and awareness campaign regarding the pandemic. In the bivariate results, both single and married respondents are less likely to observe good COVID-19 prevention practices, with singles being twice as unlikely. This may be due to the fact that the majority of singles are in the younger age bracket and have youthful exuberance, thus neglecting such preventive practices in their daily activities. Compared to younger age groups, those aged 47 to 66 were significantly more likely to engage in preventive behavior, according to the study. Those over the age of 50 with co-morbidities such as hypertension, diabetes, cancer, heart, kidney, and liver problems are the most susceptible to COVID-19. Their awareness of their susceptibility may have influenced their prudent use of preventative measures. This finding is consistent with a similar study’s conclusion that individuals under the age of 40 may not observe certain preventive measures, such as avoiding crowded areas [24]. Despite their medical and clinical backgrounds, respondents from the Faculty of Health Science were the least likely to practice good preventive measures compared to those from other faculties. This may imply overconfidence and the need to emphasize the importance of proper precautionary measures among them. Adjusting for confounders, however, revealed a decreased likelihood of COVID-19 preventive practices with age and a stronger association between adequate preventive practice and each of the following: male gender, single status, and health faculty membership. Among health care workers in Uganda, a comparable study found that females were less likely to practice COVID-19 prevention measures [26]. Regarding the influence of gender on the implementation of COVID-19 prevention measures, a survey conducted in Ethiopia [27] found that female students were more compliant than male students. Contradictory findings regarding the preventive practices of females and males highlight the importance of the potential influence of specific environments and economic status, such as employment or income, on health-seeking behaviors [28]. Importantly, a number of studies have found that sociodemographic factors, such as age, marital status, and level of education, facilitate COVID-10 prevention practices [29,30]. Similar KAP studies in universities in other regions of the world discovered that students and faculty from health science faculties adhered to COVID-19 preventive measures more closely than students and faculty from other faculties [31,32,33]. We concur with Sheek-Hussein et al. [34] that the remarkable health behaviors of students in the faculty of health sciences may be attributable to the scientific nature of their discipline, access to multiple dependable medical platforms, healthcare professionals, government media briefings, and university newsletters.

## 5. Limitations

This cross-sectional study did not examine cause-and-effect relationships, as is typical for such research. In addition, the knowledge and preventive practices of university students and faculty were self-reported online data that were susceptible to recall and information bias.

## 6. Conclusions

The study revealed that respondents take adequate and effective preventive measures. Sociodemographic factors such as age, gender, marital status, and university faculty were significant predictors of adequate and good adherence to COVID-19 preventive practices. Appropriate interventions to enhance COVID-19 prevention adherence must be context-specific and take into account the identified sociodemographic facilitators of COVID-19 prevention adherence.

### 6.1. What Is Known on This Topic

COVID-19 can be transmitted through respiratory droplets from an infected person to another when coughing and sneezing.

Preventive practices can protect against COVID-19 transmission.

### 6.2. What This Study Adds

People above the age of fifty years do have good preventive practice towards COVID-19.

Females do not practice preventive measure against COVID-19 as much as males.

## Figures and Tables

**Table 1:** Demographics of participants (*n =* 5849).

Variables	Frequency (*n*)	Percentage (%)
**Sex**		
Male	2492	42.61
Female	3351	57.29
Others	6	0.10
**Age**		
17–26	4729	80.85
27–36	852	14.57
≥37	268	4.58
Mean (SD)	23.67 ± 6.47	
**Marital Status (*n* = 5822)**		
Single	5489	94.28
Married	265	4.55
Others	68	1.17
**Level of Education**		
No formal education	19	0.32
Primary	30	0.51
Secondary	2469	42.21
Tertiary	3331	56.95

**Table 2:** Position in DUT of participants (*n =* 5849).

Variables	Frequency (*n*)	Percentage (%)
**Current position in DUT**		
Student	5623	96.14
Staff	139	2.38
Both	87	1.49
**Campus**		
Ritson Campus	1623	27.75
ML Sultan Campus	1504	25.71
Steve Biko Campus	1335	22.82
Riverside Campus	620	10.60
Indumiso Campus	583	9.97
City Campus	150	2.56
Brickfield Campus	34	0.58
**Faculty**		
Management Sciences	1766	30.19
Accounting and Informatics	1332	22.77
Engineering& the Built Environment	1064	18.19
Arts & Design	605	10.34
Applied Sciences	542	9.27
Health Sciences	540	9.23
**Current place of resident**		
Outside the university accommodation facility	4114	70.34
Within the university accommodation facility	1735	29.66

**Table 3:** Preventive practices towards COVID-19 transmission (*n =* 5849).

Variables	Frequency (*n*)	Percentage (%)
In recent days, have you gone to any crowded place?		
Yes	1501	25.66
No	4348	74.34
In recent days, have you worn a mask when leaving home?		
Yes	5730	97.97
No	119	2.03
In recent days, have you worn gloves when going out?		
Yes	2369	40.50
No	3480	59.50
In recent days, have you been using hand sanitizer regularly in public places?		
Yes	5736	98.07
No	113	1.93
In recent days, have you been washing your hands regularly after touching surfaces?		
Yes	5534	94.61
No	315	5.39
In recent days, have you been avoiding touching your mouth, eyes and nose without proper handwashing or/and using alcohol-based sanitizer?		
Yes	5139	87.86
No	710	12.14
In recent days, have you been covering your nose with tissue or cloth when you sneeze in public or at home?		
Yes	5532	94.58
No	317	5.42
In recent days, have you been observing social distancing when you are in public places		
Yes	5680	97.11
No	169	2.89
Will you accept a COVID-19 vaccine if discovered and found to be effective in preventing COVID-19?		
Yes	4249	73.73
No	1514	26.27
Do you think the university is prepared to return to function when the lockdown is eventually lifted		
Yes	4086	70.29
No	1727	29.71

**Table 4:** Overall level of preventive practices of COVID-19 transmission (*n =* 5849).

Variables	Frequency (*n*)	Percentage (%)
**Level of preventive practices of COVID-19 transmission**		
Poor preventive practices (≤5)	363	6.21
Good/adequate preventive practices (6–9)	5486	93.79

**Table 5:** Association between demographic characteristics and preventive practices of COVID-19 among participants from Durban University of Technology (DUT) (Bivariate LR) (*n =* 5849).

Variables	Level of Preventive Practices		OR (95 CI)	*p*-Value
	**Good/adequate (*n* = 5486)** **(6–9)**	**Poor (*n* = 363)** **(≤5)**		
	**Freq (%)**	**Freq (%)**		
**Current position in DUT**				
Both ^R^	83 (95.40)	4 (4.60)	*Ref*	
Student	5269 (93.70)	354 (6.30)	0.774 (0.202–2.966)	0.709
Staff	134 (96.40)	5 (3.60)	0.555 (0.226–1.365	0.200
**Sex**				
Others ^R^	4 (66.67)	2 (33.33)	*Ref*	
Male	2289 (91.85)	203 (8.15)	0.099 (0.018–0.544)	*0.008* *
Female	3193 (95.28)	158 (4.72)	0.558 (0.450–0.692)	*0.001* *
**Age**				
17–26 ^R^	4414 (93.34)	315 (6.66)	*Ref*	
27–36	813 (95.42)	39 (4.58)	9.342 (1.55–56.111)	*0.015* *
37–46	165 (97.06)	5 (2.94)	13.897 (2.257–85.586)	*0.005* *
47–56	62 (98.41)	1 (1.59)	22.00 (2.983–162.266)	*0.002* *
57–66	29 (96.67)	1 (3.33)	41.333 (2.875–594.1440)	*0.006* *
≥67	3 (60.0)	2 (40.0)	19.333 (1.327–281.597)	*0.030* *
**Marital Status**				
Others ^R^	59 (86.76)	9 (13.24)	*Ref*	
Single	5144 (93.71)	345 (6.29)	0.204 (0.076–0.551)	*0.002* *
Married	257 (96.98)	8 (3.02)	0.464 (0.228–0.946)	*0.035* *
**Level of Education**				
No formal education ^R^	18 (94.74)	1 (5.26)	*Ref*	
Primary	26 (86.67)	4 (13.33)	1.089 (0.145–8.200)	0.934
Secondary	2301 (93.20)	168 (6.80)	0.393 (0.136–1.138)	0.085
Tertiary	3141 (94.30)	190 (5.70)	0.829 (0.669–1.027)	0.085
**Faculty**				
Management Sciences ^R^	1695 (95.98)	71 (4.02)	*Ref*	
Accounting and Informatics	1237 (92.87)	95 (7.13)	1.110 (0.692–1.782)	0.664
Engineering& the Built Environment	980 (92.11)	84 (7.89)	0.606 (0.383–0.959)	*0.032* *
Arts & Design	560 (92.56)	45 (7.44)	0.543 (0.341–0.964)	*0.010* *
Applied Sciences	498 (91.88)	44 (8.12)	0.579 (0.348–0.964)	*0.035* *
Health Sciences	516 (95.56)	24 (4.44)	0.526 (0.315–0.879)	*0.014* *

* Statistically significant (*p* ≤ 0.05), ^R^ = Reference, OR = Bivariate Logistic regression.

**Table 6:** Association between demographic characteristics and preventive practices of COVID-19 among participants from Durban University of Technology (DUT) (Multivariate LR) (*n* = 5849).

Variables	Level of Preventive Practices		AOR (95 CI)	*p*-Value
	**Good/adequate (*n* = 5486)** **(6–9)**	**Poor (*n* = 363)** **(≤5)**		
	**Freq (%)**	**Freq (%)**		
**Gender**				
Others ^R^	4 (66.67)	2 (33.33)	*Ref*	
Male	2289 (91.85)	203 (8.15)	9.815 (1.721–55.959)	*0.001* *
Female	3193 (95.28)	158 (4.72)	1.692 (1.256–2.112)	*0.001* *
**Age**				
17–26 ^R^	4414 (93.34)	315 (6.66)	*Ref*	
27–36	813 (95.42)	39 (4.58)	0.059 (0.008–0.417)	*0.005* *
37–46	165 (97.06)	5 (2.94)	0.040 (0.006–0.283)	*0.001* *
47–56	62 (98.41)	1 (1.59)	0.025 (0.003–0.200)	*0.001* *
57–66	29 (96.67)	1 (3.33)	0.0015 (0.001–0.229)	*0.001* *
≥67	3 (60.0)	2 (40.0)	0.0025 (0.002–0.394)	*0.009* *
**Marital Status**				
Others ^R^	59 (86.76)	9 (13.24)	*Ref*	
Single	5144 (93.71)	345 (6.29)	6.012 (2.070–17.461)	*0.001* *
Married	257 (96.98)	8 (3.02)	1.533 (0.639–3.677)	*0.339*
**Faculty**				
Management Sciences ^R^	1695 (95.98)	71 (4.02)	*Ref*	
Accounting and Information	1237 (92.87)	95 (7.13)	0.877 (0.543–1.416)	0.591
Engineering & the Built Environment	980 (92.11)	84 (7.89)	1.474 (0.923–2.352)	0.104
Arts & Design	560 (92.56)	45 (7.44)	1.550 (0.959–2.504)	0.073
Applied Sciences	498 (91.88)	44 (8.12)	1.588 (0.947–2.661)	0.079
Health Sciences	516 (95.56)	24 (4.44)	1.721 (1.023–2.894)	*0.041* *

* Statistically significant (*p* ≤ 0.05), ^R^ = Reference, OR = Bivariate Logistic regression.

## Data Availability

The data can only be made availble by requesting through the institution.
